# Spatially Localized Two-Dimensional *J*-Resolved NMR Spectroscopy *via* Intermolecular Double-Quantum Coherences for Biological Samples at 7 T

**DOI:** 10.1371/journal.pone.0134109

**Published:** 2015-07-24

**Authors:** Chunhua Tan, Shuhui Cai, Yuqing Huang

**Affiliations:** Department of Electronic Science, Fujian Provincial Key Laboratory of Plasma and Magnetic Resonance, Xiamen University, Xiamen, China; The University of Queensland, AUSTRALIA

## Abstract

**Background and Purpose:**

Magnetic resonance spectroscopy (MRS) constitutes a mainstream technique for characterizing biological samples. Benefiting from the separation of chemical shifts and *J* couplings, spatially localized two-dimensional (2D) *J*-resolved spectroscopy (*J*PRESS) shows better identification of complex metabolite resonances than one-dimensional MRS does and facilitates the extraction of *J* coupling information. However, due to variations of macroscopic magnetic susceptibility in biological samples, conventional *J*PRESS spectra generally suffer from the influence of field inhomogeneity. In this paper, we investigated the implementation of the localized 2D *J*-resolved spectroscopy based on intermolecular double-quantum coherences (iDQCs) on a 7 T MRI scanner.

**Materials and Methods:**

A γ-aminobutyric acid (GABA) aqueous solution, an intact pig brain tissue, and a whole fish (*Harpadon nehereus*) were explored by using the localized iDQC *J*-resolved spectroscopy (iDQC*J*RES) method, and the results were compared to those obtained by using the conventional 2D *J*PRESS method.

**Results:**

Inhomogeneous line broadening, caused by the variations of macroscopic magnetic susceptibility in the detected biological samples (the intact pig brain tissue and the whole fish), degrades the quality of 2D *J*PRESS spectra, particularly when a large voxel is selected and some strongly structured components are included (such as the fish spinal cord). By contrast, high-resolution 2D *J*-resolved information satisfactory for metabolite analyses can be obtained from localized 2D iDQC*J*RES spectra without voxel size limitation and field shimming. From the contrastive experiments, it is obvious that the spectral information observed in the localized iDQC*J*RES spectra acquired from large voxels without field shimming procedure (i.e. in inhomogeneous fields) is similar to that provided by the *J*PRESS spectra acquired from small voxels after field shimming procedure (i.e. in relatively homogeneous fields).

**Conclusion:**

The localized iDQC*J*RES method holds advantage for recovering high-resolution 2D *J*-resolved information from inhomogeneous fields caused by external non-ideal field condition or internal macroscopic magnetic susceptibility variations in biological samples, and it is free of voxel size limitation and time-consuming field shimming procedure. This method presents a complementary way to the conventional *J*PRESS method for MRS measurements on MRI systems equipped with broad inner bores, and may provide a promising tool for *in vivo* MRS applications.

## Introduction

Magnetic resonance spectroscopy (MRS) is a powerful tool for investigating chemical compositions and elucidating molecular structures. It enables us to reveal valuable molecule-level information, such as chemical shifts, *J* couplings, and multiplet patterns. The spectral information, complementary to the morphological information provided by magnetic resonance imaging (MRI), is useful for analyses of biological samples [1, 2]. Due to its efficacy, MRS shows wide applications in a variety of fields [3–6]. The localized one-dimensional (1D) point-resolved spectroscopy (PRESS) is a common MRS method for practical applications with the advantage of fast acquisition [7, 8]. However, spectral congestion is generally observed in 1D PRESS spectra of biological samples because numerous resonances from various metabolites are involved in a single spectral dimension. In addition, the intrinsic magnetic susceptibility variations in biological samples generally lead to inhomogeneous line broadening in 1D PRESS spectra, even severe overlapping of spectral peaks. By separating chemical shifts and *J* couplings into two different frequency dimensions, the localized two-dimensional (2D) *J*-resolved spectroscopy (*J*PRESS) is designed by adding an indirect spectral dimension in the original 1D PRESS to alleviate the spectral congestion [9, 10]. However, the *J*PRESS method remains sensitive to field inhomogeneity caused by macroscopic magnetic susceptibility variations in biological samples, especially in the investigations of large voxels that include different components. Although inhomogeneous line broadening in the *J* coupling dimension (F1) can be refocused by the spin-echo scheme [11], the overlapping of neighboring resonances in the chemical shift dimension (F2) makes it difficult to obtain exact *J* coupling information.

Many field shimming methods have been proposed to alleviate the influence of field inhomogeneity [12, 13]. However, the field inhomogeneity in biological tissues is generally hard to eliminate by conventional shimming methods. The voxel shimming approach has been used on MRI scanners [14]. This approach is time-consuming and is not suitable for biological samples when large detection volume is concerned. The magic angle spinning (MAS) technique [15–17] provides a feasible way to remove the influence of macroscopic magnetic susceptibility variations in biological samples by fast spinning [18, 19]. In general, the MAS technique requires a specialized probe suitable for typical NMR spectrometers and is not available for MRI scanners with broad inner bores. Furthermore, fragile organic textures, such as fish eggs and viscera, cannot endure fast spinning [20]. Thus a great demand for high-resolution 2D MRS methods which can be easily adopted to standard MRI scanners for practical applications has arisen.

It has been proved that intermolecular multiple-quantum coherences (iMQCs), originating from the distant dipolar interaction among spins in different molecules, can be used to recover high-resolution NMR spectra from inhomogeneous fields [21–23]. Recently, a method (dubbed as iDQC*J*RES) based on intermolecular double-quantum coherences (iDQCs) was proposed to obtain high-resolution 2D *J*-resolved NMR spectra in inhomogeneous fields [24]. The capability of iDQC*J*RES has been tested on a common 500 MHz NMR spectrometer with samples packed in 5 mm NMR tubes. However, the feasibility of the iDQC*J*RES method for practical MRS applications on MRI scanners with broad inner bores and low magnetic field strength remains uncertain. In this study, a localized iDQC*J*RES method was investigated on a 7 T MRI scanner with different samples and different voxel sizes. Experimental results were compared with those acquired by the conventional *J*PRESS method.

## Theory

**Fig 1:**
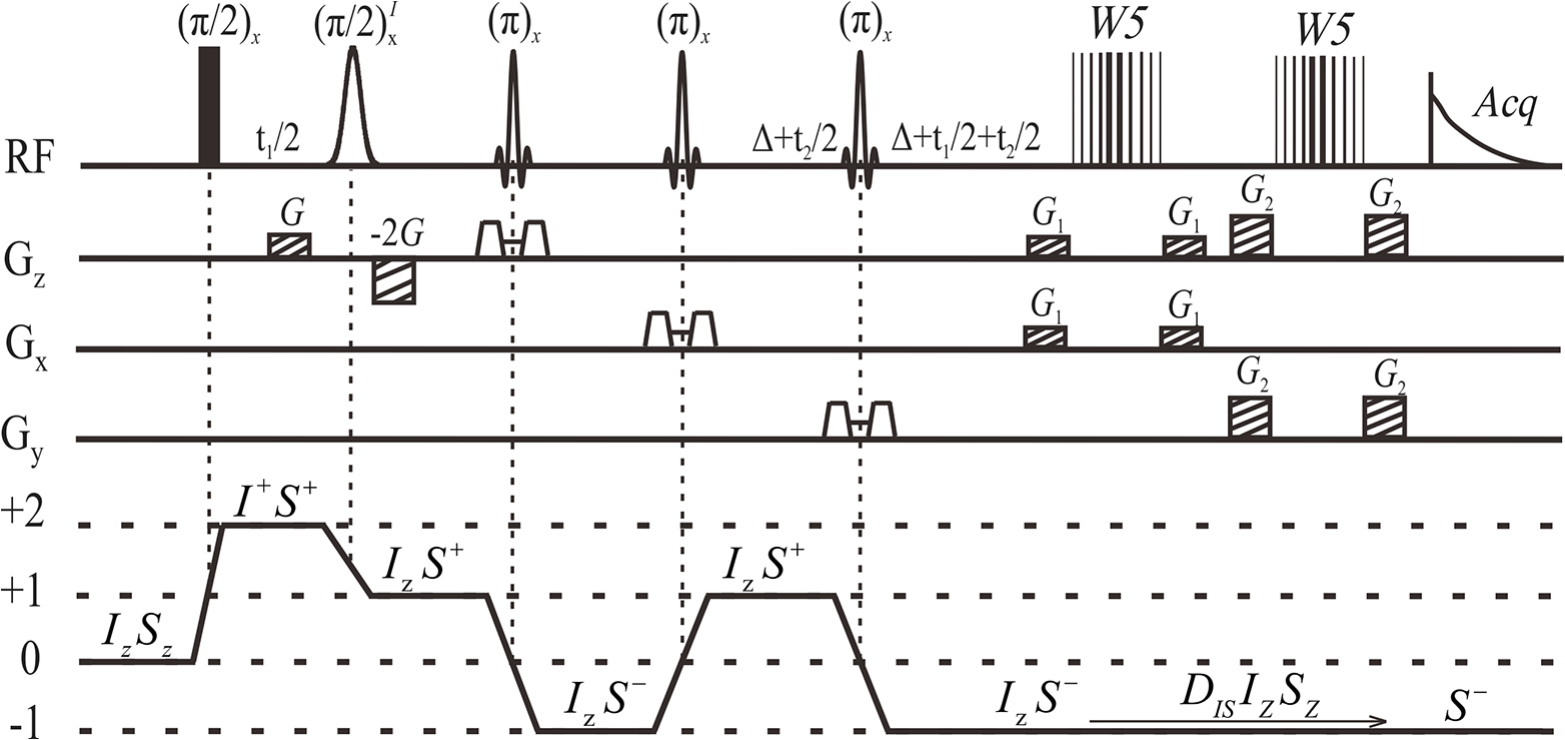
Pulse sequence diagram of the localized iDQC*J*RES. Full vertical bar is the non-selective RF pulse, Gauss-shaped pulse is solvent-selective RF pulse, Sinc-shaped pulses stand for three slice-selective refocusing *π* RF pulses, trapezoids along three orthogonal directions are slice-selective gradients, vertical-line represent “W5” binomial π pulses. *G* and -2*G* are coherence selection gradients, *G*_1_ and *G*_2_ are crasher gradients for the water suppression. The coherence transfer pathway is presented and the product operators are applied to show the coherence states of solvent *I* and solute *S* spins.

A PRESS like module [25], consisting of three slice-selective refocusing *π* RF pulses and corresponding slice-selective gradients along orthogonal directions, is integrated into the iDQC*J*RES sequence for spatial localization (Fig 1). In this localized iDQC*J*RES sequence, the PRESS like module can not only select the region of interest in the detected sample, but also refocus the resulting iDQC signals. The last slice-selective refocusing π RF pulse in the PRESS like module is inserted into the middle of the delay interval (2Δ) to preserve the desired signals before the distant dipolar interaction takes effect. Therefore, the non-selective π RF pulse used in the non-localized iDQC*J*RES can be omitted in the localized iDQC*J*RES sequence. Water suppression is a prerequisite for measurements of biological samples. Different from water suppression modules used in the original PRESS and *J*PRESS sequences, the double gradient echo W5 module implemented right before acquisition period is used in the localized iDQC*J*RES sequence to suppress the water signal [26, 27]. In this water suppression module, the crusher gradients are applied along the *x*, *y* and z directions. The first π/2 RF pulse is non-selective, and the second RF pulse (π/2)^*I*^ is selective for the water proton. A pair of linear coherence selection gradients (CSGs) with an area ratio of 1:-2 are employed along the *z* direction to select the desired coherence transfer pathway 0 → +2 → +1 → −1. Two indirect evolution periods, *t*_1_ and *t*_2_, are used for the desired signal evolution. Consider a homogeneous solution consisting of *I* (corresponding to solvent) and *S* (corresponding to solute) components, where *I* is an isolated single spin-1/2 system and *S* is an AX spin-1/2 system that includes *S*_*k*_ and *S*_*l*_ spins coupled by a *J*_*kl*_ scalar interaction. The evolution of two-spin order term for the desired signal from the localized iDQC*J*RES sequence can be understood intuitively by the product operator analysis as following,

$$I_{z}S_{z}\overset{\;(\pi /2)\;}{\to }\frac{1}{4}I^{+}S^{+}\;\left(t_{1}/2\right)\overset{\;{\left(\pi /2\right)}^{I}\;}{\to }\frac{1}{8}I_{z}S^{+}\;\left(t_{2}/2\right)\overset{\;\left[\left(\pi )-(\pi )-(\pi \right)\right],\;D_{IS}I_{Z}S_{z}\;}{\to }\frac{1}{8}S^{-}\;\left(t_{1}/2+t_{2}/2+t_{3}\right),$$ (1)

where *D*_*IS*_ *I*_z_ *S*_z_ represents the distant dipolar interaction for iDQC between solvent and solute spins. According to the iMQC treatment [28], high temperature approximation is abandoned and the two spin term *I*_*z*_ *S*_*z*_ is the start point for the signal evolution. The localized iDQC*J*RES sequence starts with a non-selective π/2 RF pulse, and the iDQC term *I* ^+^ *S* ^+^ is selected by the CSGs and evolved during the first evolution periods *t*_1_/2. After that, the second (*π*/2)^*I*^ RF pulse selective for *I* spin transforms *I* ^+^ into (0.5*I* ^+^ − 0.5*I* ^−^ − *I*_*z*_) and only the term *I*_*z*_ *S* ^+^ is persevered by the CSGs. Then the PRESS like module localizes the region of interest in the sample and overturns the coherence order from *I*_*z*_ *S* ^+^ to *I*_*z*_ *S* ^−^. Finally, the spin term *I*_*z*_ *S* ^−^ evolves into observable signal by the distant dipolar interaction during the evolution period *t*_1_/2+*t*_2_/2+*t*_3_. In this sequence, two indirect evolution periods, *t*_1_ and *t*_2_, are used and each is divided into two equal parts for the desired signal evolution. For the indirect evolution period *t*_1_, *I* ^*+*^ *S* ^*+*^ (iDQC term) evolution is involved in the first *t*_1_/2 and *S*¯ evolution is involved in the second *t*_1_/2, thus only the field inhomogeneity and *J* coupling are preserved in the F1 dimension. For the indirect evolution period *t*_2_, *I*_z_ *S* ^*+*^ evolution is involved in the first *t*_2_/2 and *S*¯ evolution is involved in the second *t*_2_/2, thus only the *J* coupling is observed in the F2 dimension. Before the acquisition period *t*_3_, the distant dipolar interaction takes effects and transfers *I*_*z*_ *S*¯ into observable *S*¯ for signal acquisition. The field inhomogeneity effect remains in the F3 dimension. Since the double gradient echo W5 module right before acquisition only acts to suppress the water signal and does not influence the desired coherence transfer pathway and the desired solute signals, we ignore it in the theoretical analysis.

According to the previous report [24], the observable transverse magnetization of the *S*_*k*_ spin from the localized iDQC*J*RES sequence in an inhomogeneous field is given as

$$\begin{array}{l} M_{+}^{S_{k}}(t_{1},t_{2},t_{3})=\frac{M_{0}^{S}\pi (0.5t_{1}+0.5t_{2}+t_{3})}{4\tau_{d}^{I}}\text{cos}\left[\pi J_{kl}(2\Delta +t_{1}+t_{2}+t_{3})\right]e^{-i\left(\omega_{I}+\Delta B_{I}(r)\right)t_{1}/2}e^{i\left(\omega_{S_{k}}+\Delta B_{S_{k}}(r)\right)t_{3}}\\ \;=\frac{M_{0}^{S}\pi (0.5t_{1}+0.5t_{2}+t_{3})}{8\tau_{d}^{I}}\left\{\begin{array}{l} e^{i\left[-(\omega_{I}/2+\Delta B_{I}(r)/2-\pi J_{kl})t_{1}+\pi J_{kl}t_{2}+(\omega_{S_{k}}+\Delta B_{S_{k}}(r)+\pi J_{kl})t_{3}\right]}e^{i2\Delta \pi J_{kl}}\\ -e^{i\left[-(\omega_{I}/2+\Delta B_{I}(r)/2+\pi J_{kl})t_{1}-\pi J_{kl}t_{2}+(\omega_{S_{k}}+\Delta B_{S_{k}}(r)-\pi J_{kl})t_{3}\right]}e^{-i2\Delta \pi J_{kl}} \end{array}\right\} \end{array}$$ (2)

where *ω*_*m*_ is the frequency offset of spin *m* (*m* = *I*, *S*_*k*_, *S*_*l*_) in the rotating frame free of field inhomogeneity, and Δ*B*_*m*_(***r***) is the inhomogeneous deviation of the magnetic field at the location of a particular *m* spin; $M_{0}^{S}$ and $M_{0}^{I}$ are equilibrium magnetizations per unit volume of *S* and *I* spins, respectively; *μ*_0_ is the vacuum magnetic permeability, and *γ* is the gyromagnetic ratio. Eq (2) provides a quantitative expression of the 3D iDQC*J*RES signal between the solute spin *S*_*k*_ and the solvent spin *I*. In this equation, the terms $e^{i(\pm \pi J_{kl}t_{2})}$ represent the *J* coupling information along the F2 dimension, and the terms $e^{i(\omega_{S_{k}}+\Delta B_{S_{k}}(r)\pm \pi J_{kl})t_{3}}$ represent the evolution information of the solute along the F3 dimension, including chemical shift, field inhomogeneity, and *J* coupling. The terms $e^{i(-\omega_{I}/2-\Delta B_{I}(r)/2\pm \pi J_{kl})t_{1}}$ are the iDQC terms along the F1 dimension, including chemical shift and field inhomogeneity of the solvent, and *J* coupling of the solute. If the spectrometer reference frequency coincides with the resonance frequency of *I* spin in *B*_0_, i.e. *ω*_*I*_ = 0, the 3D iDQC*J*RES signal will split into two peaks and locate at $\left(\Delta B_{I}(r)/2-\pi J_{kl},\;-\pi J_{kl},\;\omega_{S_{k}}+\Delta B_{S_{k}}(r)+\pi J_{kl}\right)$ and $\left(\Delta B_{I}(r)/2+\pi J_{kl},\;\pi J_{kl},\;\omega_{S_{k}}+\Delta B_{S_{k}}(r)-\pi J_{kl}\right)$. In the localized iDQC*J*RES sequence, the dipolar correlation distance between *S* and *I* spins is inversely proportional to the area of CSGs, that is *d*_*c*_ = *π*/(*γGδ*) [29]. Generally, this distance is much smaller than the sample size, thus the magnetic field within the distance between *S* and *I* spins only varies slightly, and Δ*B*_*I*_(***r***) is considered to be equal to $\Delta B_{S_{k}}(r)$. A shearing process on the F1-F3 plane is carried out to remove inhomogeneous line broadening along the F3 dimension. The frequency location for the signal in the sheared 3D spectrum becomes $\left(\Delta B_{I}(r)/2-\pi J_{kl},\;-\pi J_{kl},\;\omega_{S_{k}}+3\pi J_{kl}\right)$ and $\left(\Delta B_{I}(r)/2+\pi J_{kl},\;\pi J_{kl},\;\omega_{S_{k}}-3\pi J_{kl}\right)$. A projection of the sheared 3D spectrum onto the F2-F3 plane produces a desired high-resolution 2D *J*-resolved spectrum, and the signal locates at $\left(-\pi J_{kl},\;\omega_{S_{k}}+3\pi J_{kl}\right)$ and $\left(\pi J_{kl},\;\omega_{S_{k}}-3\pi J_{kl}\right)$. A clockwise rotation of this 2D iDQC*J*RES projection spectrum along F2 = 0 can separate chemical shifts and *J* couplings, resulting in peak positions at $\left(-\pi J_{kl},\;\omega_{S_{k}}\right)$ and $\left(\pi J_{kl},\;\omega_{S_{k}}\right)$, the same as the signal observed in a conventional 2D *J*PRESS spectrum.

## Methods and Materials

All experiments were executed at 293 K using a Varian (Palo Alto, CA, USA) 7 T small animal magnetic resonance scanner with a 160 mm inner bore diameter and a 63/95 mm quad birdcage coil. The scanner was equipped with a gradient coil system producing a maximum gradient strength of 40 G/cm. The quad birdcage coil was well tuned to preserve high signal sensitivity. For comparison, the *J*PRESS sequence [30] for localized 2D *J*-resolved spectra was utilized as a reference scheme in our experiments on aqueous solution, pig brain tissue, and fish. For the localized iDQC*J*RES experiments, a 4-step phase cycling was applied: the phases for the first π/2 pulse, the second (π/2)^*I*^ RF pulse, and the receiver were (*x*, *y*,-*x*,-*y*), (*x*, *x*,-*x*,-*x*), and (*x*,-*x*,-*x*, *x*), respectively. The methods and experiments on biological samples were carried out in accordance with the approved guidelines. All biological samples (a whole fish and an intact pig brain tissue) used in our experiments were approved by the Institutional Review Board at Xiamen University, Xiamen, China.

### Aqueous Solution

A γ-aminobutyric acid (GABA) aqueous solution (250 mM) filled in a plastic bottle with a volume of 68 cm^3^ was used to demonstrate the feasibility of the localized iDQC*J*RES sequence on the MRI scanner. Prior to spectral experiments, fast spin-echo images on coronal and axial orientations of the plastic bottle were acquired to show the localized regions. The magnetic field was deliberately degraded by altering the Z1 shimming coil current to produce broad peaks. The full width at half maximum (FWHM) of the water peak at 4.80 ppm was 180 Hz, and the full width at 10% maximum was 535 Hz. In this inhomogeneous field, the *J*PRESS and localized iDQC*J*RES sequences were applied to the same voxel with a size of 18 × 18 × 18 mm^3^. In addition, the magnetic field was shimmed using the standard shimming procedure provided in the MRI scanner, and then a *J*PRESS experiment and a localized iDQC*J*RES experiment on a voxel size of 6 × 6 × 6 mm^3^ under this shimmed field were performed for comparison. For the localized iDQC*J*RES experiments, the width of the π/2 hard RF pulse was 90 μs, the solvent-selective (π/2)^*I*^ RF pulse had a Gaussian shape with a width of 6.0 ms. The power levels of the π/2 hard RF pulse and solvent-selective π/2 pulse were 200 W and 250 mW, respectively. The parameters for the crusher gradients in the water suppression module were *G*_1_ = 9.6 G/cm, *G*_2_ = 26.9 G/cm, and *δ*′ = 3.0 ms. The width of sinc-shaped π pulses was 2.0 ms, and the parameters for the three slice-selective gradients were set to *G*_*x*_ = *G*_*y*_ = *G*_*z*_ = 0.29 G/cm with a duration of 2.0 ms for the 18 × 18 × 18 mm^3^ voxel, and *G*_*x*_ = *G*_*y*_ = *G*_*z*_ = 0.90 G/cm with a duration of 2.0 ms for the 6 × 6 × 6 mm^3^ voxel. Other parameters for the localized iDQC*J*RES experiments were as follows: the pulse repetition time TR = 2.0 s, the echo time (TE) 2Δ = 54 ms, the acquisition time = 100 ms, the average number = 4, and 10 × 30 × 600 points were acquired with spectral widths of 100 Hz × 50 Hz × 3000 Hz (F1 × F2 × F3) in 40 min. The localized iDQC*J*RES 3D data were processed using our custom-written program on MATLAB 7.11. For *J*PRESS experiments, the parameters for the spatial localization were set to *G*_*x*_ = 1.27 G/cm with a duration of 1.0 ms, *G*_*y*_ = *G*_*z*_ = 0.29 G/cm with a duration of 2.0 ms for the 18 × 18 × 18 mm^3^ voxel, and *G*_*x*_ = 3.82 G/cm with a duration of 1.0 ms, *G*_*y*_ = *G*_*z*_ = 0.90 G/cm with a duration of 2.0 ms for the 6 × 6 × 6 mm^3^ voxel. The variable power and optimized relaxation delays (VAPOR) module was used for water suppression. The TR/TE was 2000/15 ms, the acquisition time was 100 ms, the average number was 4, and 30 × 600 points were acquired with spectral widths of 50 Hz × 3000 Hz (F1 × F2) in 4 min.

### Pig Brain Tissue

A sample of intact pig brain tissue was applied to show the capability of the localized iDQC*J*RES sequence on biological tissues. The pig brain tissue was purchased from a local retailer named Fujian New Hua Du Supermarket Co., LTD. (24°44'N, 118°09'E), and the item number for this sample was FJNQ896563328. The pig brain tissue was carefully packed in a fresh bag and kept in a fresh layer of fridge at 5°C for about one hour before the experiments. Prior to spectral measurements, fast spin-echo images on axial and coronal orientations were acquired with TR/TE = 2500/40 ms and imaging matrix = 256 × 256 in circa 5 min. The iDQC*J*RES experiment was performed without any field shimming. In this experiment, the widths of π/2 hard pulse and solvent-selective (π/2)^*I*^ RF pulse were 98 μs and 5.9 ms, respectively. The parameters of the CSGs and the WS module were the same as those used for the GABA aqueous solution experiment. The parameters of spatial localization were *G*_*x*_ = *G*_*y*_ = *G*_*z*_ = 0.29 G/cm with a duration of 2.0 ms for the 18 × 18 × 18 mm^3^ voxel. The TR/TE was 2000/36 ms, the acquisition time was 105 ms, the average number was 16, and 9 × 30 × 650 points were acquired with spectral widths of 100 Hz × 50 Hz × 3000 Hz (F1 × F2 × F3) in 144 min. A *J*PRESS experiment on the same voxel under the same field condition was performed for comparison. As a reference, a *J*PRESS experiment with a small voxel of 6 × 6 × 6 mm^3^ under a relatively homogeneous field was performed after the standard shimming procedure. The VAPOR module was used for water suppression. The *J*PRESS experiments were acquired with TR/TE = 2000/15 ms, 16 average number, 30 × 650 points for spectral widths of 50 Hz × 3000 Hz (F1 × F2) in 16 min.

### A Whole Fish

To show the applicability of the localized iDQC*J*RES sequence on real biological samples with integrated organism, we performed a postmortem study on a whole fish (*Harpadon nehereus*). The fish sample was purchased from the same local retailer where we bought the pig brain tissue, with an item number of FJDS042108187. This fish sample was originally supplied by Dongshan fishery (23°33'N, 117°17'E). The purchased fish was carefully packed in a fresh bag and preserved in a fresh layer of fridge at 5°C for about one hour before experiments. The standard shimming procedure was executed to optimize the field homogeneity. A fast spin-echo MRI experiment was carried out to display inner structures of the fish in axial and coronal planes. For the iDQC*J*RES experiment, the width of the π/2 hard pulse was 111 μs and the width of solvent-selective (π/2)^*I*^ RF pulse was 6.2 ms. The parameters of the CSGs and the WS module were the same as those used in the aqueous solution experiment. The parameters of spatial localization were *G*_*x*_ = *G*_*y*_ = *G*_*z*_ = 0.33 G/cm with a duration of 2.0 ms for the 16 × 16 × 16 mm^3^ voxel. The TR/TE was 2000/36 ms, the acquisition time was 120 ms, the average number was 32, and 9 × 25 × 720 points were acquired with spectral widths of 100 Hz × 50 Hz × 3000 Hz (F1 × F2 × F3) in 240 min. Two *J*PRESS experiments on the voxels of 16 × 16 × 16 mm^3^ and 6 × 6 × 6 mm^3^ were performed, respectively, with TR/TE = 2000/15 ms, 32 average number, and 25 × 720 points for spectral widths of 50 Hz × 3000 Hz (F1 × F2) in 26.7 min.

## Results and Discussion

**Fig 2:**
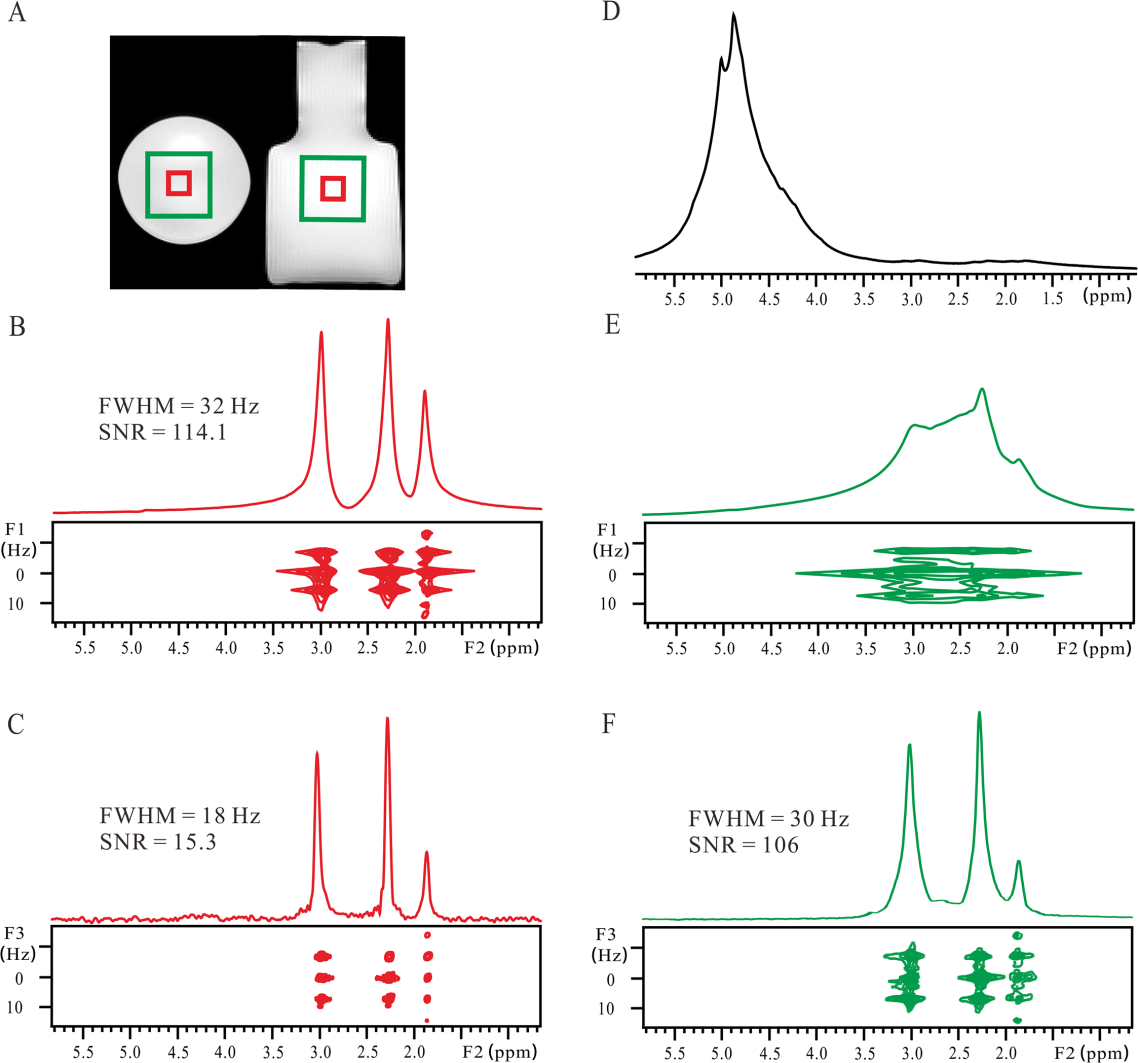
Results of the GABA aqueous solution. (A) Spin echo images of the GABA solution filled into a plastic bottle. A large voxel of 18 × 18 × 18 mm^3^ is marked by a green rectangle, and a small voxel of 6 × 6 × 6 mm^3^ is marked by a red rectangle. (B) Conventional 2D *J*PRESS spectrum after a clockwise rotation of 45° and its projection along the F2 axis acquired from the small voxel in a relatively homogeneous field after the field shimming procedure. (C) Localized iDQC*J*RES spectrum and its 1D *J*-decoupled projection along the F3 axis acquired from the small voxel under the same field homogeneity as (B). (D) Conventional 1D non-localized spectrum acquired in an inhomogeneous field with a 180 Hz FWHM of the water peak at 4.8 ppm. (E) Conventional 2D *J*PRESS spectrum after a clockwise rotation of 45° and its projection along the F2 axis acquired from the large voxel in an inhomogeneous field. (F) Localized iDQC*J*RES spectrum and its 1D *J*-decoupled projection along the F3 axis acquired from the large voxel in the same inhomogeneous field as (E).

The feasibility of the localized iDQC*J*RES on recovering high-resolution 2D *J*-resolved spectra from inhomogeneous fields at the 7 T MRI scanner is verified by the GABA aqueous solution experiment (Fig 2). The fast spin-echo images of the GABA aqueous solution filled in a plastic bottle at coronal and axial sections are given to show the localized regions (Fig 2A). After a clockwise rotation of 45°, the 2D *J*PRESS spectrum acquired from the voxel of 6 × 6 × 6 mm^3^ after the standard shimming procedure is presented as a reference (Fig 2B). Owing to the broad inner bore of the 7 T MRI scanner, it is hard to keep the magnetic field absolutely homogeneous. Thus the FWHM of the peak at 2.3 ppm along the F2 dimension of the 2D *J*PRESS spectrum remains 32 Hz (Fig 2B). The 2D *J*-resolved information can be obtained, and three coupled peaks of GABA are observed along the F2 dimension, and the related *J* coupling constants and multiplet patterns are presented in the F1 dimension. A 2D *J*-resolved spectrum obtained from the localized iDQC*J*RES method with the same voxel size and field condition is also presented (Fig 2C). Benefiting from the immunity to field inhomogeneity of the iDQC*J*RES method, the FWHM of the peak at 2.3 ppm along the F2 dimension of the 2D localized iDQC*J*RES spectrum reaches to 18 Hz. It is obvious that the signal to noise ratio (SNR) of this spectrum is lower than that of the conventional *J*PRESS spectrum (Fig 2B). To make a clear comparison, we performed SNR calculations on these two experiments. The SNR is calculated by dividing the intensity of the peak at 3.01 ppm by the standard deviation of noise signals in the region between 5.0 and 5.5 ppm along the 1D projection [31]. The SNR is 15.3 for Fig R2C, while it is 114.1 for Fig R2B. So the SNR of the 2D localized iDQC*J*RES spectrum only presents 13.4% of that of the conventional 2D *J*PRESS spectrum.

However, when the magnetic field is deliberately deshimmed, the valuable spectral information will be lost in conventional spectra. A conventional 1D non-localized spectrum of the sample was acquired without water suppression to show the field condition (Fig 2D). The FWHM of the water peak at 4.8 ppm is 180 Hz, and the information of chemical shifts and *J* couplings is erased by inhomogeneous line broadening. Similarly, the conventional *J*PRESS spectrum also suffers from the influence of field inhomogeneity. It is difficult to extract satisfactory information from the 2D *J*PRESS spectrum acquired from the voxel of 18 × 18 × 18 mm^3^ under this inhomogeneous field (Fig 2E). All signal peaks stretch along the F2 axis due to inhomogeneous line broadening. Although the field inhomogeneity can be refocused by spin echo scheme along the F1 dimension, the overlapping among neighboring peaks obscures *J* coupling measurement. However, under the same inhomogeneous field and from the same voxel, a high-resolution 2D *J*-resolved spectrum can be obtained by using the localized iDQC*J*RES method (Fig 2F). Compared to the conventional 2D *J*PRESS spectrum (Fig 2E), the spectral resolution along the chemical shift dimension (F3) is significantly improved in the 2D localized iDQC*J*RES spectrum, and the FWHM for the peak at 2.3 ppm is reduced to 30 Hz. In addition, *J* coupling constants and multiplet patterns are explicitly shown in the F2 dimension. The spectral features provided in the 2D localized iDQC*J*RES spectrum in the inhomogeneous field are the same as those obtained from the conventional 2D *J*PRESS spectrum in the relatively homogeneous field. Furthermore, the SNR of the 2D iDQC*J*RES spectrum acquired from the relatively large voxel of 18 × 18 × 18 mm^3^ in the inhomogeneous field is 106.5 (Fig 2F), close to the SNR of the conventional 2D *J*PRESS spectrum acquired from the voxel of 6 × 6 × 6 mm^3^ under the relatively homogeneous field (Fig 2B). Thus, a relatively large voxel can partially compensate for the weakness of the localized iDQC*J*RES method on SNR. It is notable that the echo time used in the localized iDQC*J*RES experiments is longer than that used in the conventional *J*PRESS experiments (54 ms for iDQC*J*RES, 15 ms for *J*PRESS). Long echo time for the conventional *J*PRESS will lead to signal decay caused by the transverse relaxation (*T*_2_ relaxation). Therefore, the default echo time of 15 ms on the MRI scanner was used in the *J*PRESS experiments to preserve maximal *J*PRESS signals. In the case of iMQC MRS experiments, since the distant dipolar interaction needs some time to take effect (the so-called “demagnetizing time”), the signal grows first and then decays following *T*_2_ relaxation [29]. We performed an arrayed experiment to seek optimal echo time for maximal iDQC*J*RES signal. It turns out that the echo time of 54 ms is optimal for the iDQC*J*RES experiments.

**Fig 3:**
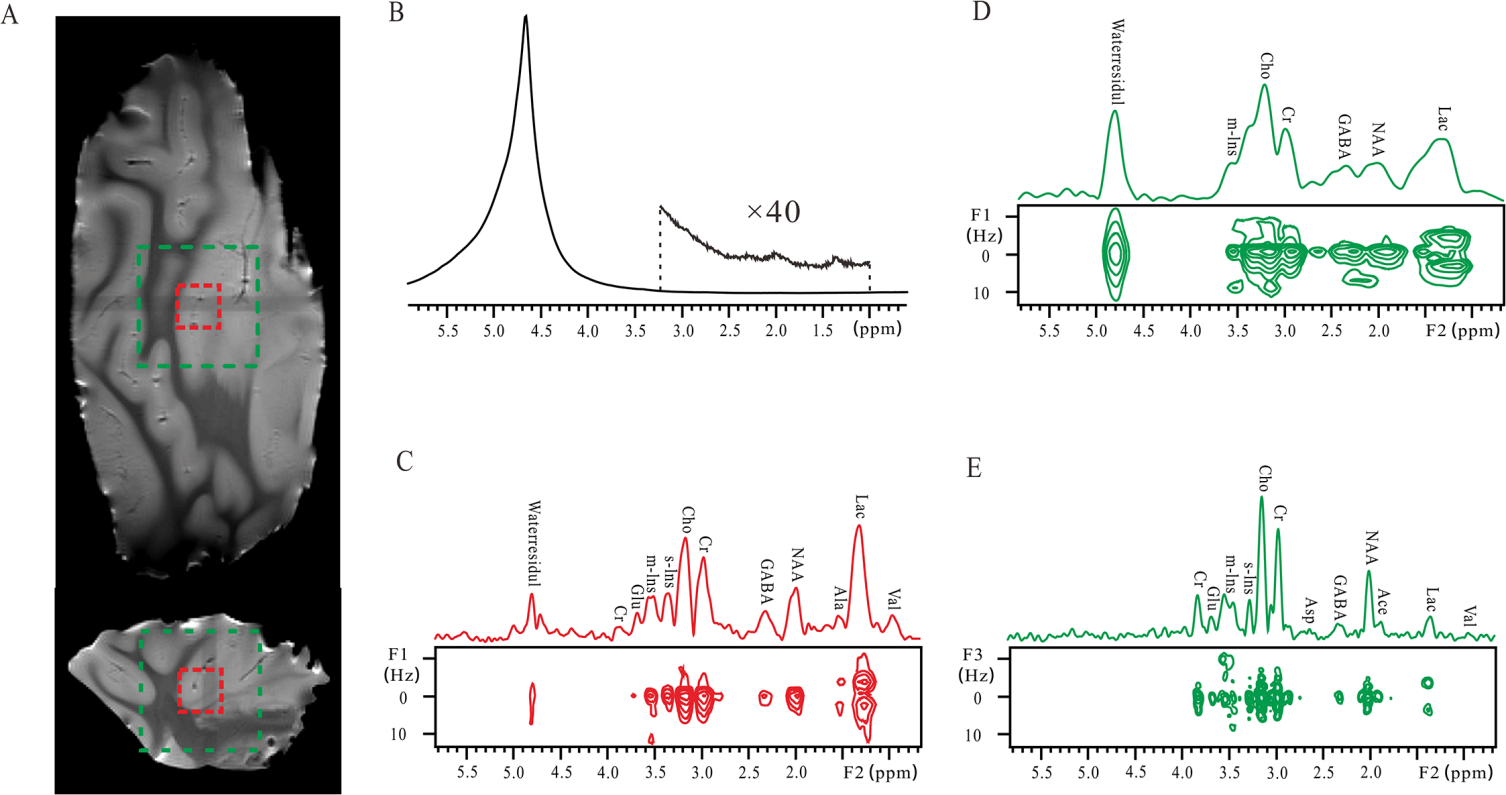
Results of an intact pig brain tissue. (A) Spin echo images of the sample. A large voxel of 18 × 18 × 18 mm^3^ is marked by a green dashed rectangle, and a small voxel of 6 × 6 × 6 mm^3^ is marked by a red dashed rectangle. (B) Conventional 1D non-localized spectrum and the expanded region for metabolites. (C) Conventional 2D *J*PRESS spectrum after a clockwise rotation of 45° and its projection along the F2 axis acquired from the small voxel after the field shimming procedure. (D) Conventional 2D *J*PRESS spectrum after a clockwise rotation of 45° and its projection along the F2 axis acquired from the large voxel without the field shimming procedure. (E) 2D localized iDQC*J*RES spectrum and its 1D *J*-decoupled projection along the F3 axis acquired from the large voxel without the field shimming procedure.

The experimental results of the intact pig brain tissue are presented in Fig 3. The fast spin-echo images of the brain tissue at coronal and axial sections are given to show voxel positions (Fig 3A). Due to the field inhomogeneity caused by intrinsic macroscopic magnetic susceptibility variations, hardly any spectral information can be obtained from the conventional 1D non-localized spectrum (Fig 3B). The conventional 2D *J*PRESS spectrum and its 1D projection along the F2 dimension acquired from the voxel of 6 × 6 × 6 mm^3^ are shown in Fig 3C. In MRS studies of biological tissues, the field inhomogeneity is directly dependent on the selected voxel size [32]. Therefore the field homogeneity in the *J*PRESS experiment on a small voxel of 6 × 6 × 6 mm^3^ can be well after the field shimming procedure. The FWHM of choline (Cho) at 3.20 ppm is 39 Hz (Fig 3C) and the major metabolite peaks are observed and assigned. The *J* coupling information of some metabolites can be measured, such as D-lactic acid (Lac) at 1.34 ppm and alanine (Ala) at 1.53 ppm. However, when a relatively large voxel of 18 × 18 × 18 mm^3^ is selected, the field homogeneity decreases remarkably due to the variations of magnetic susceptibility among various structures of the brain tissue. The resulting 2D *J*PRESS spectrum is influenced by inhomogeneous line broadening and the desired spectral information for metabolite analyses is lost (Fig 3D). The peaks are overlapped along the F2 dimension and the *J* coupling information is hard to identify along the F1 dimension. Although a large voxel is beneficial to signal intensity, the aggravated field inhomogeneity makes the application of the conventional *J*PRESS method on large voxels challenging.

The localized 2D iDQC*J*RES spectrum acquired from the large voxel of 18 × 18 × 18 mm^3^ without any field shimming procedure is given in Fig 3E. Compared to the conventional 2D *J*PRESS spectrum, the spectral resolution is significantly improved. Taking the Cho peak at 3.18 ppm for an example, its FWHM is 30 Hz in this localized iDQC*J*RES spectrum (Fig 3E), 100 Hz in the conventional 2D *J*PRESS spectrum of the voxel of 18 × 18 × 18 mm^3^ without the field shimming (Fig 3C), and 39 Hz in the conventional 2D *J*PRESS spectrum of the voxel of 6 × 6 × 6 mm^3^ after the field shimming (Fig 3D). Clearly, the localized iDQC*J*RES spectrum can yield satisfactory spectral information, and this information is even better than that provided in the conventional *J*PRESS spectrum acquired from the small voxel in the shimmed field. A considerable number of metabolite signals are resolved and assigned [33] in the localized iDQC*J*RES spectrum. To make a comparison of the results obtained by the localized iDQC*J*RES method from the large voxel and by the conventional *J*PRESS method from the small voxel, we list the ^1^H chemical shifts, multiplet patterns, and *J* coupling constants extracted from the two spectra (Fig 3C and 3E) in Table 1. Twelve peaks could be assigned to 11 metabolites in the localized iDQC*J*RES spectrum from the large voxel, while 11 peaks could be assigned to 10 metabolites in the conventional *J*PRESS spectrum from the small voxel.

**Table 1:** Comparison of spectral results of an intact pig brain tissue obtained by localized iDQC*J*RES and conventional *J*PRESS methods.

		^1^H chemical shifts (ppm)	Multiplet patterns**	*J* coupling constants (Hz)
Metabolite	Group	iDQC*J*RES/*J*PRESS*	iDQC*J*RES/*J*PRESS	iDQC*J*RES/*J*RESSS
Valine (Val)	-CH_3_	1.07/0.98	n.o. *** /n.o.	n.o./n.o.
Lactate (Lac)	-CH-C**H**_3_	1.33**/**1.30	d**/**d	7.0**/**6.7
Alanine (Ala)	-CH_3_	n.o./1.53	n.o.**/**d	n.o.**/**6.5
Acetate (Ace)	-CH_3_	1.89**/** n.o.	s**/** n.o.	—**/**n.o.
N-Acetyl aspartate	-CH_3_	2.02**/**1.98	s**/**s	—**/**—
(NAA)				
γ-Aminobutyric acid	-C**H**_2_-CH_2_	2.31**/**2.28	t**/**s	7.4**/**n.o.
(GABA)				
Aspartate (Asp)	-CH	2.75**/**n.o.	s**/**n.o.	—**/**n.o.
Creatine (Cr)	-CH_3_	3.0**/**2.98	s**/**s	—**/**—
	-CH_2_	3.85**/**3.90	s**/**s	—**/**—
Choline (Cho)	-CH_3_	3.18**/**3.20	s**/**s	—**/**—
*Scyllo*-inositol (s-Ins)	-CH	3.30**/**3.31	s**/**s	—**/**—
*Myo*-inositol (m-Ins)	-CH	3.50**/**3.52	t**/**t	10.2**/**10.0
Glutamate/Glutamine	-CH	3.69/3.65	s/s	—**/**—
(Glu/Gln)				

* The left and right values in the following lists are from the iDQC*J*RES spectrum of the large voxel and the *J*PRESS spectrum of the small voxel, respectively.

** Multiplet patterns are defined as: singlet (s), doublet (d), triplet (t), quartet (q), double doublet (dd), and multiplet (m).

*** n.o. = not observable.

In a previous study, a localized MRS method based on intermolecular single-quantum coherences has been used to obtain 1D spectrum with enhanced resolution on pig brain tissues [34]. The spectral resolution therein was not enough for observing *J* coupling splitting, and only chemical shifts could be observed. In the localized iDQC*J*RES spectrum, chemical shifts and *J* couplings are provided along two separate dimensions. Chemical shifts directly point to metabolite assignments and *J* couplings aid metabolite identification. It can be noticed that the apparent signal intensity of Lac is decreased in the localized iDQC*J*RES spectrum (Fig 3E) compared to that in the conventional *J*PRESS spectrum (Fig 3D). Similar result was observed in the previous MRS study on the brain tissue [32, 34, 35]. The main reason is that the lipid signal at 1.25 ppm generally overlaps with the Lac signal at 1.30 ppm due to the insufficient spectral resolution. The contribution of lipid signal to the signal intensity can be observed in the *J*PRESS spectrum with a short echo time. Because a long echo time was adopted in the iDQC*J*RES experiment, the decay of lipid signal became severe due to its short transverse relaxation time, hence a relatively weak Lac signal free of the interference of lipid signal was obtained in the iDQC*J*RES spectrum.

**Fig 4:**
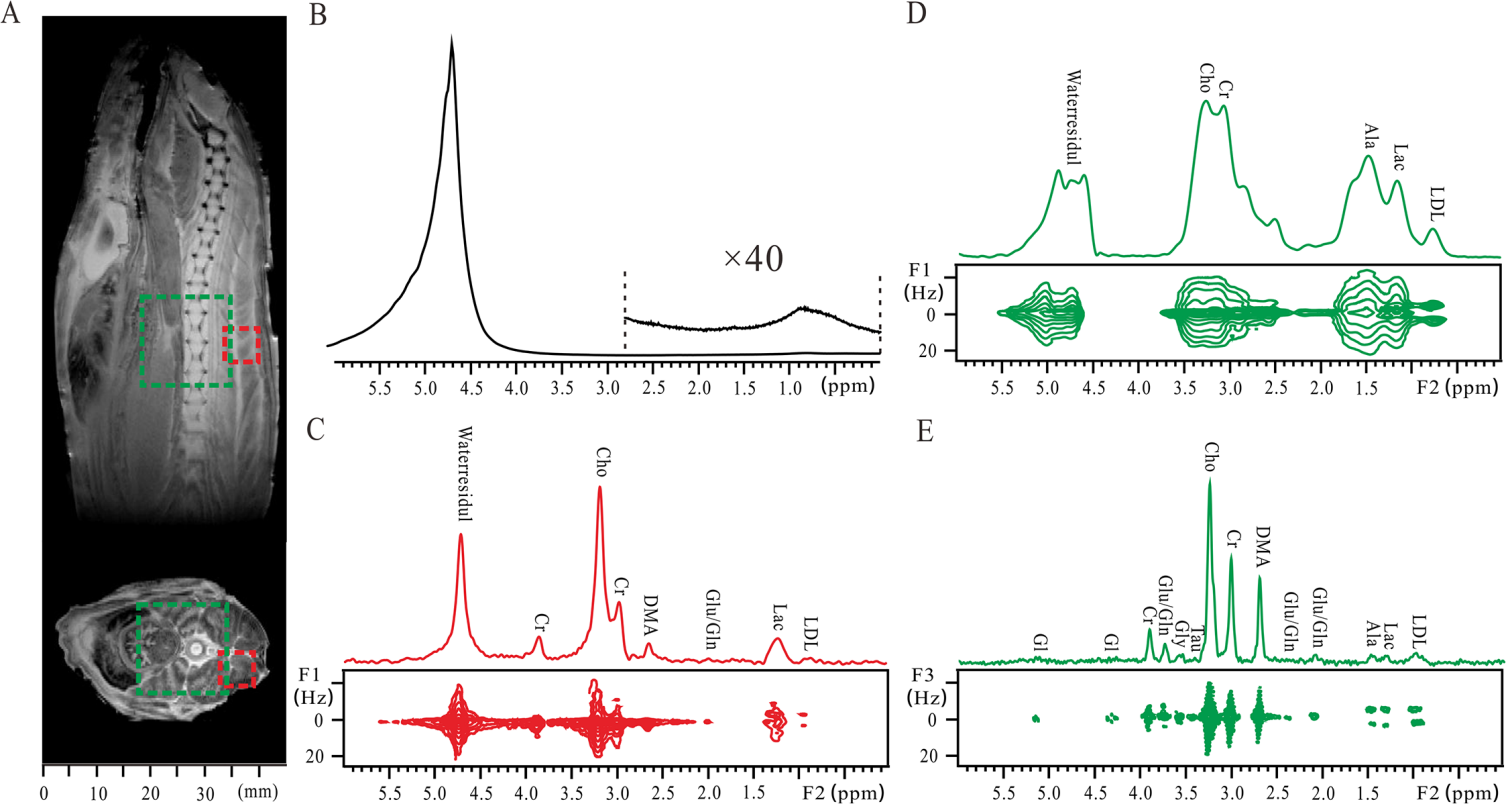
Results of a whole fish (*Harpadon nehereus*). (A) Spin echo images of the fish. A large voxel of 16 × 16 × 16 mm^3^ containing the fish spinal cord is marked by a green dashed box, and a small voxel of 6 × 6 × 6 mm^3^ containing the fish tissue is marked by a red dashed box. (B) Conventional 1D non-localized spectrum and its expanded region for metabolites. (C) Conventional 2D *J*PRESS spectrum after a clockwise rotation of 45° and its projection along the F2 axis acquired from the small voxel after the field shimming procedure. (D) Conventional 2D *J*PRESS spectrum after a clockwise rotation of 45° and its projection along the F2 axis acquired from the large voxel after the field shimming procedure. (E) 2D localized iDQC*J*RES spectrum and its 1D *J*-decoupled projection along the F3 axis acquired from the large voxel without the field shimming procedure.

The ability of the localized iDQC*J*RES method on enhancing spectral resolution from strongly structured biological tissues is exhibited by the experiments of a whole fish (*Harpadon nehereus*) (Fig 4). The coronal and axial spin-echo images of the fish are displayed in Fig 4A. The length of the fish is about 200 mm and only part of the axial image is given. A large voxel of 16 × 16 × 16 mm^3^ containing the fish spinal cord is marked by a green dashed box in the two images, while a small voxel of 6 × 6 × 6 mm^3^ containing only the fish flesh is marked by a red dashed box in these images. Hardly any spectral information can be obtained from the conventional 1D non-localized spectrum due to the field inhomogeneity (Fig 4B). When the conventional *J*PRESS method is applied to the small voxel, the field inhomogeneity can be partially removed by field shimming, and a 2D *J*PRESS spectrum with acceptable resolution can be obtained (Fig 4C). The FWHM of Cho at 3.22 ppm is 38 Hz and some metabolites can be observed. The *J* coupling information of the metabolites, such as the methyl group of low-density lipoprotein (LDL) at 0.93 ppm and lactate (Lac) at 1.34 ppm, can be extracted along the F1 dimension. Because of the prolate shapes and surrounding bone structures, the field shimming for the fish spinal cord region is generally challenging [36]. Thus, when the large voxel containing the fish spinal cord is selected, the quality of the resulting 2D *J*PRESS spectrum remarkably decreases even after field shimming (Fig 4D). The FWHM of the water peak at 4.80 pm is 120 Hz. Most metabolite peaks are overlapped and lost along the F2 dimension, and only the *J* coupling splitting of the methyl group of low-density lipoprotein (LDL) at 0.75 ppm can be observed along the F1 dimension.

A 2D localized iDQC*J*RES spectrum, acquired from the voxel of 16 × 16 × 16 mm^3^ containing the fish spinal cord and without field shimming, provides 2D *J*-resolved information with enhanced spectral resolution (Fig 4E). The FWHM of Cho at 3.25 ppm is 28 Hz along the F3 dimension in this 2D spectrum, better than 38 Hz along the F2 dimension of the 2D *J*PRESS spectrum acquired from the small voxel after field shimming. A considerable number of metabolite signals are identified and assigned. Benefiting from the spectral resolution enhancement, some weak metabolite signals invisible in the *J*PRESS spectrum (Fig 4D) can be observed in the iDQC*J*RES spectrum (Fig 4E), such as alanine (Ala) at 1.48 ppm. The assignment of the observed peaks according to literature [20, 37] is given in Fig 4. To make a clear comparison between the results obtained by the iDQC*J*RES method from the large voxel without field shimming and by the conventional *J*PRESS method from the small voxel after field shimming, we list the ^1^H chemical shifts, multiplet patterns, and *J* coupling constants in Table 2. Fourteen peaks are assigned to 11 metabolites in the localized iDQC*J*RES spectrum, while 7 peaks are assigned to 6 metabolites in the conventional *J*PRESS spectrum. Note that the observed peaks in the spectra acquired from the same fish with different methods have some differences. For example, glutamate/glutamine (Glu/Gln) at 3.75 ppm and glycine at 3.55 ppm are present in the iDQC*J*RES spectrum while absent in the conventional *J*PRESS spectrum. This may be attributed to the complex circumstance, magnetic susceptibility gradient between the fish tissue and the air in abdominal cavity, and intrinsic magnetic susceptibility variations in fish itself among muscle tissues, viscera and bones. The difference in the voxel selection may pose another possible reason.

**Table 2:** Comparison of 1H NMR spectral results of a whole fish (*Harpadon nehereus*) obtained using iDQC*J*RES and conventional *J*PRESS methods.

		^1^H chemical shifts (ppm)	Multiplet patterns**	*J* coupling constants (Hz)
Metabolite	Group	iDQC*J*RES/*J*PRESS*	iDQC*J*RES/*J*PRESS	iDQC*J*RES/*J*RESSS
low-density lipoprotein	C**H**_3_-(CH_2_)_n_-	0.95/0.93	d**/**d	6.6/6.3
(LDL)				
Lactate (Lac)	-CH-C**H**_3_	1.33**/**1.25	d/s	7.1**/**n.o.***
Alanine (Ala)	-CH_3_	1.45/n.o.	d/n.o.	6.9**/**n.o.
Glutamate/Glutamine	-CH_3_	2.08/2.02	m/s	n.o.**/** —
(Glu/Gln)	-CH_2_	2.38/ n.o.	s/ n.o.	n.o./n.o.
	-CH_2_	3.75/n.o.	t/n.o.	6.2**/**n.o.
Dimethylamine (DMA)	-CH_3_	2.69/2.65	s/s	—/—
Creatine (Cr)	-CH_3_	3.00**/**3.00	s**/**s	—**/**—
	-CH_2_-	3.90**/**3.86	s**/**s	—**/**—
Choline (Cho)	-CH_3_	3.25/3.22	s/s	—**/**—
Taurine (Tau)	-CH_2_	3.40/n.o.	t/n.o.	6.6/n.o.
Glycine (Gly)	-CH_2_	3.55/n.o.	s/n.o.	—/n.o.
Glyceryl (Gl)	-CH_2_	4.30/n.o.	s/n.o.	—/n.o.
	-CH_2_	5.13/n.o.	s/n.o.	—/n.o.

* The left and right values in the following lists are from the iDQC*J*RES spectrum of the large voxel and the *J*PRESS spectrum of the small voxel, respectively.

** Multiplet patterns are defined as: singlet (s), doublet (d), triplet (t), quartet (q), double doublet (dd), and multiplet (m).

*** n.o. = not observable.

All above results show that the localized iDQC*J*RES method can be applied for direct measurement of biological samples to obtain high-resolution *J*-resolved information, without the limitation of voxel size and field shimming requirement. However, the localized iDQC*J*RES method also has disadvantages in signal sensitivity and experimental time in contrast to the conventional *J*PRESS method. On the aspect of signal sensitivity, the use of large voxel can partially compensate for the low signal intensity. Besides, high sensitivity probes [38] and parallel coils [39] on MRI scanners may be useful for the improvement of signal intensity for the localized iDQC*J*RES method. On the aspect of acquisition time, the localized iDQC*J*RES experiment takes longer acquisition time than the conventional *J*PRESS since 3D acquisition is required. The spatial encoding scheme [40] may be applied to the *t*_1_ period of the localized iDQC*J*RES sequence, so *t*_1_ increments can be sampled in a single scan, and the acquisition time can be shortened to the level of the conventional 2D *J*PRESS sequence. It is obvious that both of the conventional *J*PRESS and the localized iDQC*J*RES method have their own advantages and drawbacks in practical applications. The conventional *J*PRESS method is useful for the measurements of metabolites in a specific small area of interest under a relatively homogeneous magnetic field. The localized iDQC*J*RES method is more useful for the measurements of metabolites in a relatively large area with field inhomogeneity, such as the lesion area in a large animal or human body [41]. In practical applications, there is no technique applicable to all circumstances, and the localized iDQC*J*RES method may provide a complementary way to the conventional *J*PRESS method for MRS measurements of biological samples on MRI systems.

## Conclusion

In this work, the implementation of the localized iDQC*J*RES method on a 7 T MRI scanner is studied. The experiment on a GABA aqueous solution reveals the feasibility of the localized iDQC*J*RES method on refocusing inhomogeneous line broadening on the 7 T MRI scanner. Spatially localized applications on biological samples are demonstrated on an intact pig brain tissue and a whole fish. The spectral information observed in the localized iDQC*J*RES spectra acquired under inhomogeneous fields (due to large voxel and no field shimming) is similar to that provided by the *J*PRESS spectra acquired in relatively homogeneous fields (due to small voxel and field shimming). Our experimental observations clearly illuminate the advantages of the localized iDQC*J*RES method for 2D MRS applications on MRI systems with broad inner bores. This method presents an alternative to the conventional *J*PRESS method for MRS measurements on biological samples, and may be a promising tool for *in vivo* MRS applications.
